# Sulforaphane’s Nuclear Factor Erythroid 2-Related Factor 2 (Nrf2)-Dependent and -Independent Mechanism of Anti-SARS-CoV-2 Activity

**DOI:** 10.35534/jrbtm.2024.10010

**Published:** 2024-06-24

**Authors:** Ziqi Yan, Weifeng Liang, Lingxiang Zhu, Ivana Kreso, Venesa Romero, Melisa Smith, Yin Chen

**Affiliations:** Department of Pharmacology and Toxicology, School of Pharmacy, University of Arizona, Tucson, AZ 85721, USA

**Keywords:** SARS-CoV-2, Nrf2, Lung, Sulforaphane, MA30, M^Pro^

## Abstract

It is well established that Nrf2 plays a crucial role in anti-oxidant and anti-inflammatory functions. However, its antiviral capabilities remain less explored. Despite this, several Nrf2 activators have demonstrated anti-SARS-CoV-2 properties, though the mechanisms behind these effects are not fully understood. In this study, using two mouse models of SARS-CoV-2 infection, we observed that the absence of Nrf2 significantly increased viral load and altered inflammatory responses. Additionally, we evaluated five Nrf2 modulators. Notably, epigallocatechin gallate (EGCG), sulforaphane (SFN), and dimethyl fumarate (DMF) exhibited significant antiviral effects, with SFN being the most effective. SFN did not impact viral entry but appeared to inhibit the main protease (M^Pro^) of SARS-CoV-2, encoded by the Nsp5 gene, as indicated by two protease inhibition assays. Moreover, using two Nrf2 knockout cell lines, we confirmed that SFN’s antiviral activity occurs independently of Nrf2 activation *in vitro*. Paradoxically, *in vivo* tests using the MA30 model showed that SFN’s antiviral function was completely lost in Nrf2 knockout mice. Thus, although SFN and potentially other Nrf2 modulators can inhibit SARS-CoV-2 independently of Nrf2 activation in cell models, their Nrf2-dependent activities might be crucial for antiviral defense under physiological conditions.

## 1. Introduction

Nrf2 is a critical transcription factor in the regulation of cellular defense mechanisms against oxidative and electrophilic stress. It regulates the expression of numerous antioxidant and phase II detoxification enzymes by binding to the antioxidant response element (ARE) in the promoter regions of target genes. Under normal conditions, Nrf2 is kept in the cytoplasm bound to its inhibitor, Keap1 (Kelch-like ECH-associated protein 1), which facilitates Nrf2’s degradation by the ubiquitin-proteasome pathway [1,2]. Upon exposure to oxidative stress, chemopreventive agents, or other electrophile attacks, Nrf2 dissociates from Keap1 and translocates into the nucleus, where it induces the transcription of various protective genes involved in cellular defense, detoxification, and the maintenance of redox homeostasis [1,2]. This mechanism is pivotal in protecting cells from environmental insults and contributes to the prevention of diseases such as cancer, neurodegenerative diseases, and inflammatory conditions [3–5].

Sulforaphane (SFN), epigallocatechin gallate (EGCG), dimethyl fumarate (DMF), bardoxolone methyl (CDDO-ME), and 4-octyl itaconate (4-OI) are diverse compounds known for their ability to modulate the Nrf2 pathway. SFN, naturally found in cruciferous vegetables, is a potent Nrf2 activator, enhancing antioxidant responses [6]. EGCG, the major catechin in green tea, indirectly activates Nrf2 by inhibiting its negative regulator, Keap1, thus promoting antioxidant protection [7]. DMF, approved for treating multiple sclerosis [8], activates Nrf2 by modifying cysteine residues on Keap1 [9], enhancing cellular resilience against oxidative damage. CDDO-ME, a synthetic triterpenoid, robustly induces Nrf2, leading to heightened expression of cytoprotective genes [10]. Lastly, 4-OI, a derivative of itaconic acid, activates Nrf2 by alkylating critical cysteine residues on Keap1 [11]. These compounds underscore the therapeutic potential of targeting the Nrf2 pathway in various disease conditions.

The COVID-19 pandemic, caused by SARS-CoV-2 (β-coronavirus), has highlighted the contagious nature of such viruses, primarily spreading through respiratory transmission [12]. With over 760 million reported infections and a death toll surpassing 6.9 million, according to the World Health Organization at the time of this report, it stands as the most severe pandemic since the Spanish flu over a century ago. Moreover, in less than 20 years, three β-coronaviral outbreaks (SARS-CoV, MERS-CoV, and SARS-CoV-2) have reached endemic or pandemic levels, positioning coronaviral infection as a significant and ongoing public health threat. Nrf2 has been investigated for its function in SARS-CoV-2 infection and COVID-19 therapeutic development [13–16] due to previous research supporting its anti-inflammatory and antiviral functions [14], which help mitigate symptoms of respiratory viral infections. Furthermore, it has been shown that SARS-CoV-2 suppresses the Nrf2 signaling pathway [17,18] suggesting a potential viral evasion strategy and further supporting the potential role of Nrf2 in host resistance to SARS-CoV-2. To this end, SFN [19,20], EGCG [21], DMF [17], CDDO-ME [22], and 4-OI [17] have been reported to exhibit antiviral effects in several SARS-CoV-2 infection models. However, these studies were conducted independently, making it difficult to compare their activities. Most importantly, while some studies explicitly demonstrated the indispensable role of Nrf2 activation [20], others did not directly indicate its involvement [17,21,22]. Our study was initially designed to re-evaluate these Nrf2 activators for their anti-SARS-CoV-2 activity and whether or not they are Nrf2 dependent within unified models. Ultimately, in this study, we discovered an Nrf2-independent antiviral mechanism of SFN, as well as the potential contribution of Nrf2 activation to anti-SARS-CoV-2 defense under physiological conditions.

## 2. Materials and Methods

### 2.1. Chemical Compounds, Cell Culture and Viruses

SFN (T8281), GC376 (T5188) were obtained from TargetMol (Wellesley Hills, MA). CDDO-ME (Cat. 11883), 4-OI (Cat. 25374), DMF (Cat. 14714) were purchased from Cayman Chemical (Ann Arbor, MI). EGCG (Cat. 324880) was obtained from MilliporeSigma (Burlington, MA, USA). The stock solutions were prepared in DMSO. 293T, Vero-E6, Vero-TMPRSS2-T2A-ACE2 (VTA) cells were obtained from American Type Culture Collection (ATCC, Manassas, VA, USA) and cultured in DMEM supplemented with 10% fetal bovine serum (FBS), and 1% penicillin/streptomycin. The VTA cells were maintained with the addition of 10 μg/mL of puromycin. Wild-type A549 (A549-WT), NRF2 KO A549 (A549-NRF2 KO), wild-type BEAS2B (BEAS2B-WT), NRF2 KO BEAS2B (BEAS2B-NRF2 KO) cell lines were generous gifts from Dr. Donna D Zhang’s lab (The University of Arizona, AZ, USA) and grown in DMEM supplemented with 10% FBS. The KO lines were cultured with the addition of 55 μM of β-Mercaptoethanol. Due to the low availability of ACE2 in A549 and BEAS2B cells, all WT and KO cell lines were transduced with adenoviral hACE2 (BEI resources, NR-52390, MOI = 1) at forty-eight hours before SARS-CoV-2 infections. This transduction had no impact on cell viability (data not shown). A549-hACE cell (Cat. NR-53821) was obtained from BEI Resources and cultured in DMEM supplemented with 10% FBS. The construction of the SARS-CoV-2 Spike-pseudotyped lentivirus was based on a published protocol [23]. SARS-CoV-2, isolate USA_WA1/2020, was generously supplied by Jennifer Harcourt from Natalie J. Thornburg Lab. This strain used for all of the *in vitro* experiments. SARS-CoV-2 Omicron BA.4.6 strain (Cat. NR-58715) was obtained from BEI Resources and propagated in VTA cells in DMEM supplemented with 2% FBS. Cells were lysed by frozen-thaw and supernatants were collected after 72 hours post-infection (hpi), stored in aliquots at −80 °C and titrated using plaque assays. SARS-CoV-2-MA30 strain [24] was provided by Dr. Stanley Perlman (The University of Iowa) and Dr. Nicholas J Maness’s lab (Tulane University) under separated MTAs. All SARS-CoV-2 related work was conducted in a biological safety cabinet in a biosafety level 3 laboratory at University of Arizona based on an approved protocol.

### 2.2. RNA Extraction, cDNA Synthesis and Real-Time Quantitative PCR (qPCR)

Total RNA was extracted from tissues using RNeasy Mini Kit (QIAGEN, Germantown, MD, USA) according to the manufacturer’s protocol. cDNA was prepared from 1000 ng of total RNA and was then further diluted to 100 ul with 10mM Tris for the following procedures. Two microliters of diluted cDNA were subjected to amplification of selected genes by real-time quantitative PCR using SYBR Green PCR Master Mix by a Veriti^®^ Thermal Cycler (Thermo Fisher, Grand Island, NY, USA). Primers were used at 0.25 μM. The primer is designed by us using the Primer 3 software. The relative mRNA amount in each sample was calculated based on the △△Ct method using housekeeping gene β- Actin. Results were calculated as fold induction over control [25]. Primers are listed in Table 1.

### 2.3. Antibodies and Western Blot

Anti-NP (R019) was purchased from SinoBiological (Houston, TX, USA). Anti-ACE2 (AF933) was obtained from R&D. Anti-His tag (2365s), anti-NSP5 (51661) were from Cell Signaling Technology (Danvers, MA, USA). Anti-NRF2 (13032), anti-HO-1 (136960) and anti-β-ACTIN (47778) were from Santa Cruz Biotechnology (Dallas, TX, USA). Equal protein loading was confirmed using anti-β-Actin. Total cellular proteins were collected based on the methods described previously [26]. Equal protein loading was confirmed using anti-β-actin. The experiment was repeated at least three times.

### 2.4. Antiviral Activity Assay

Vero-E6 cells were seeded in a 24-well plate at 4 × 10^4^ cells per well for antiviral compound validation. After 72 h, the cells reached 100% confluency and were pre-treated with either vehicle (DMSO) or the compounds at the indicated concentrations for 1 h. The cells were then infected with SARS-CoV-2 at approximately 50 PFU for 1 h. Cell cultures were covered with a semi-solid overlay medium (1% *w*/*v* methylcellulose in DMEM supplemented with 5% FBS) and incubated for three days. After the removal of the overlay medium, the cells were fixed in 10% neutral buffered formalin (NBF) for 30 min and stained with a 0.9% *w*/*v* crystal violet solution, and the plaques were counted. The percentage of inhibition was calculated using the formula [1 – (V_compound_/V_DMSO_)] × 100%, where V_compound_ and V_DMSO_ refer to the virus titers in the presence of the tested compound and DMSO, respectively.

### 2.5. Luciferase Reporter Assay

A549-hACE2 cells were pre-treated with the indicated concentrations of SFN for 1 h, followed by infection with the SARS-CoV-2 Spike-pseudotyped lentivirus at an MOI of 1. Forty-eight hpi, the cells were lysed and subjected to luciferase activity measurement.

### 2.6. Auto Docking

The three-dimensional structure of M^Pro^ (PDB ID: 7nxh) was retrieved from the Protein Data Bank (http://www.rcsb.org). The 3D conformation of SFN (CID_9577379) was acquired from PubChem (https://pubchem.ncbi.nlm.nih.gov), a repository providing data on the pharmacological activities of small molecules. Both the protein and ligand underwent preparation steps for molecular docking using AutoDock Tools (ADT) (version 1.5.7). The protein structure was enhanced by adding hydrogen atoms, while water molecules were removed. Similarly, ligand structures were augmented with hydrogen atoms, and root and torsional information was detected. A grid box, centered on the catalytic receptor residue Cysteine 145 of M^Pro^, was generated using AutoGrid 4 (version 4.2.6). The iBabel (version 5.0.2) program was used to convert the PDB file format. AutoDock 4 (version 4.2.6) was employed for executing the molecular docking simulations. Finally, the visualization of docking results was accomplished using PyMOL (Version 2.5.0).

### 2.7. Recombinant Protein Purification, Fluorescence Resonance Energy Transfer (FRET) Assay, and M^Pro^ Protease Activity Assay

The purified recombinant M^Pro^ and its substrates were kindly supplied by Dr. Hongmin Li’s lab (The University of Arizona, AZ, USA). The cloning and purification of M^Pro^ and the positive substrates containing its cleavage site were previously described [27,28]. The substrate used for the FRET assay contained a CFP-YFP pair. Briefly, 0.2 μM SARS-CoV-2 M^Pro^ was incubated with different concentrations of compounds or DMSO in assay buffer containing 20 mM Tris pH 8.0, 100 mM NaCl, 1 mM DTT, and 1 mM EDTA at room temperature for 30 min. 20 μM of the CFP-YFP substrate was added to initiate the enzyme reaction. The proteolytic reaction was carried out at 30 °C in a BioTek Synergy HI microplate reader with filters for excitation at 435 nm and emission at 475/530 nm. Reactions were monitored for 2 h and read every 2 min. The IC_50_ values were calculated by fitting a nonlinear regression using GraphPad Prism 9 software. For M^Pro^ protease activity assay, 2 μM M^Pro^ was incubated for 1 h with different concentrations of each inhibitor (DMSO, SFN, Nirmatrelvir, GC376) in reaction buffer (20 mM Tris pH 8.0, 100 mM NaCl, 1 mM DTT, and 1 mM EDTA). Subsequently, the His-tagged substrate was added at a concentration of 5 μM and further incubated for 1 h, followed by Western blot analysis.

### 2.8. Mouse Models of SARS-CoV-2 Infection

Animal studies were conducted based on protocols approved by the Institutional Animal Care and Use Committee at the University of Arizona. Mice were housed in the Animal Care facility at the University of Arizona under standard conditions. Adult C57BL/6-WT and Nrf2 KO mice, aged 6–8 weeks, were used for this study. The mice were briefly anesthetized with isoflurane and infected intranasally with the specified amount of virus in a total volume of 100 μL of DPBS. Animal weight and health were monitored daily. For the SFN experiments, mice were treated intranasally with 10 mg/kg of SFN or the vehicle DMSO daily. Treatment commenced one day prior to viral infection. At day 5 post-infection (dpi), the mice were euthanized by an overdose of isoflurane, and lung tissues were collected. Lung tissue homogenate supernatants were titered using a plaque assay in VTA cells. Viral titers were quantified as PFUs per milligram (mg) of protein concentration in the supernatants.

### 2.9. Statistical Analysis

Experimental groups were compared using a two-sided Student’s *t* test, with significance level set as *p* < 0.05. When data were not distributed normally, significance was assessed with the Wilcoxon matched-pairs signed-ranks test, and *p* < 0.05 was considered to be significant.

## 3. Results

### 3.1. Nrf2 Deficiency Had a Significant Impact on Mouse Models of SARS-CoV-2 Infection

**Figure 1.**
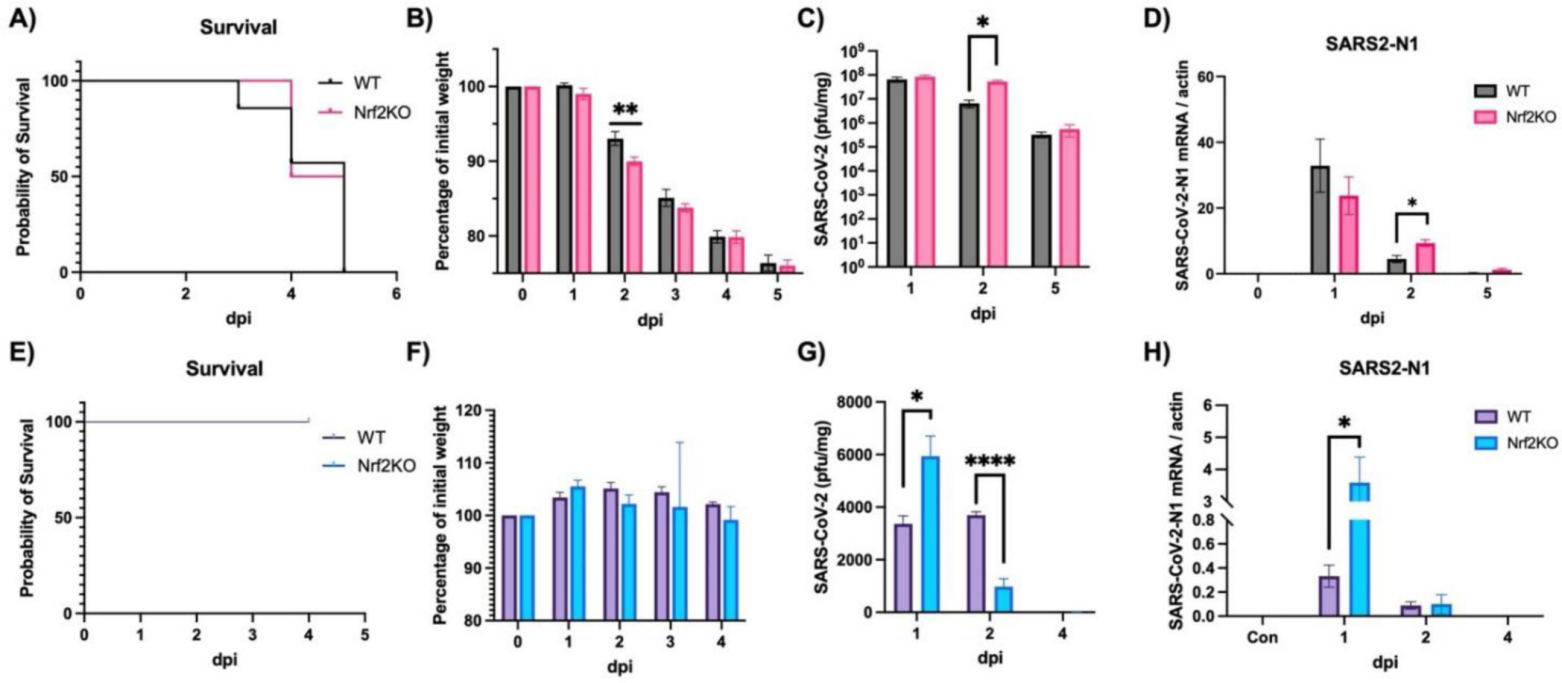
Effects of Nrf2 deficiency on survival, body weight and viral production. WT: wild-type mice. Nrf2 KO: Nrf2 Knockout mice. (**A**–**D**) Mice were intranasally infected with 10^5^ PFU/mouse MA30 and samples were collected at dpi as designated in the figures. (**A)** Survival, (**B**) body weight, (**C**) lung viral titer, (**D**) viral N1 gene expression by qPCR. Actin: control. (**E**–**H**) Mice were intranasally infected with 10^5^ PFU/mouse Omicron BA.4.6 and samples were collected at dpi as designated in the figures. (**E**) Survival, (**F**) body weight, (**G**) lung viral titer, (**H**) viral N1 gene expression by qPCR. Actin: control. *: *p* < 0.05. **: *p* < 0.01. ****: *p* < 0.0001. (*n* = 8–10 for survival and body weight, *n* = 4–6 for other analyses).

To investigate the role of Nrf2 in anti-SARS-CoV-2 defense, we utilized two different mouse models: a mouse-adapted SARS-CoV-2 model (MA30) and a mouse model of human Omicron-BA.4.6 infection. The MA30 model is lethal at 10^5 PFU/mouse (Figure 1A) with a significant drop in body weight starting at 2 dpi (Figure 1B) (Note: lower doses do not cause mortality, data not shown). In contrast, the BA.4.6 model is not lethal (Figure 1E) and shows no significant changes in body weight (Figure 1F). The absence of Nrf2 did not affect overall survival in both models (Figure 1A,E). However, in the MA30 model, body weight was significantly lower in Nrf2 KO mice at 2 dpi compared to WT mice (Figure 1B). Consistently, both viral titer (Figure 1C) and viral gene expression (N1) (Figure 1D) were significantly higher at 2 dpi in the MA30 model. In the BA.4.6 model, however, there was a significant increase in viral titer at 1 dpi followed by a rapid decrease at 2 dpi (Figure 1G) in the absence of Nrf2. In this model, viral gene expression (N1) was consistently elevated at 1 dpi in Nrf2 KO mice, but there was no difference between WT and Nrf2 KO mice at 2 and 4 dpi (Figure 1H), presumably due to the rapid decline in lung viral load in this model. This discrepancy between the MA30 and BA.4.6 models may be attributed to the nature of these two models. MA30 is considered a replicating virus in mice, as demonstrated by a markedly higher lung viral titer (up to 1 × 10^8^ PFU/mg) (Figure 1C), while BA.4.6 does not appear to replicate in the mouse lung, exhibiting much lower lung viral titers (up to 6 × 10^3^ PFU/mg) despite the same initial viral inoculation. Additionally, despite being a replicating virus in the mouse, the lung titer of MA30 also rapidly decreased before the final mortality at 5 dpi.

**Figure 2.**
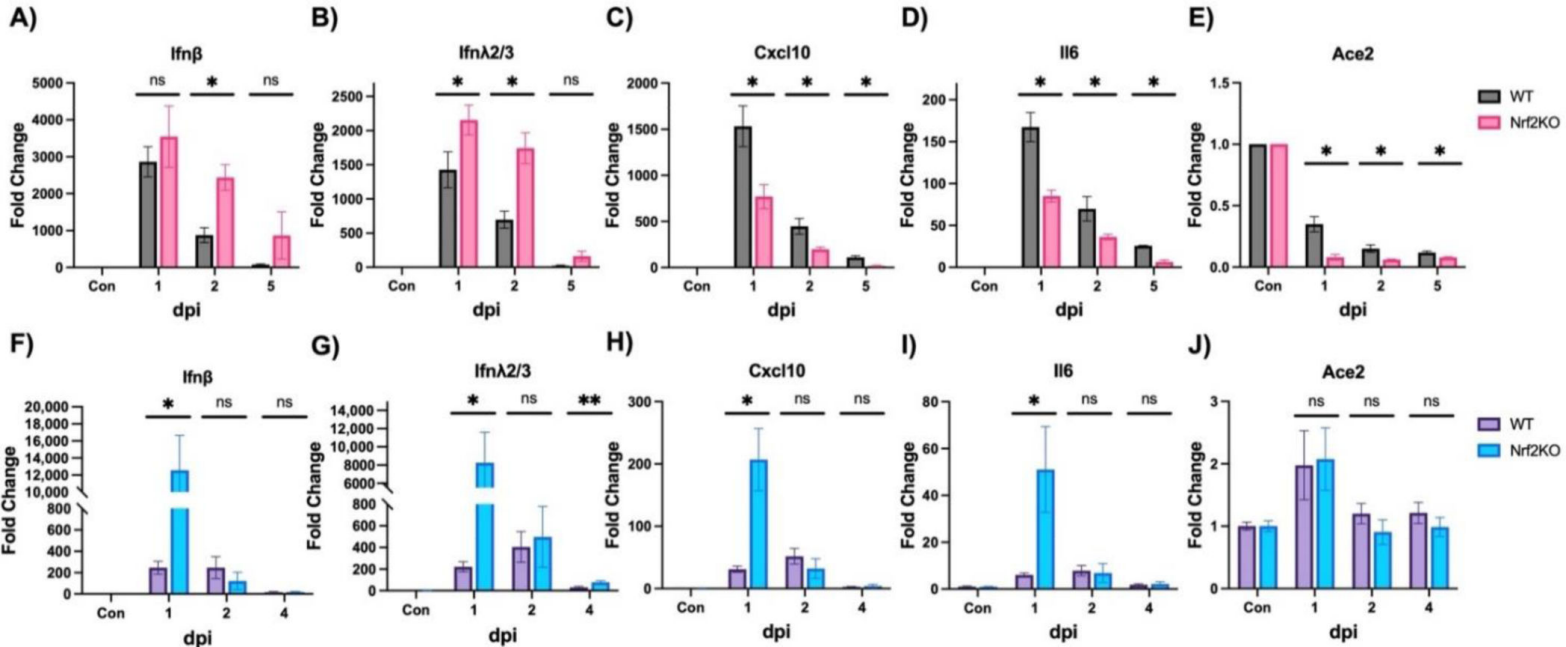
Effects of Nrf2 deficiency on cytokine gene expression. WT: wild-type mice. Nrf2 KO: Nrf2 Knockout mice. (**A**–**E**) Mice were intranasally infected with 10^5^ PFU/mouse MA30 and samples were collected at dpi as designated in the figures. Cytokine expressions were measured by qPCR. Actin was used as a control and data are presented as fold induction. (**F**–**J**) Mice were intranasally infected with 10^5^ PFU/mouse Omicron BA.4.6 and samples were collected at dpi as designated in the figures. *: *p* < 0.05. **: *p* < 0.01. ns: not significant. *n* = 4–6.

We also measured various lung gene expressions in these two infection models. In the MA30 model, Nrf2 deficiency resulted in higher expressions of *Ifnβ* (Figure 2A) and *λ2/3* (Figure 2B) at 2 dpi, which was consistent with the increased viral titer and viral gene expression (Figure 1C,D). However, expressions of *Cxcl10* (Figure 2C) and *Il6* (Figure 2D) were lower in Nrf2 KO mice. In the BA.4.6 model at 1 dpi, Nrf2 deficiency significantly enhanced expressions of Type I/III interferon (*Ifnβ*, *λ2/3*) (Figure 2F,G), *Cxcl10* (Figure 2H), and *Il6* (Figure 2I), aligning with the viral titer and viral gene expression (Figure 1G,H). Since the viral receptor *Ace2* was reported to be repressed at mRNA level by SARS-CoV-2 infection in various cell models [29], we tested if this phenomenon also occurred *in vivo*. Indeed, MA30 infection markedly repressed *Ace2* gene expression (Figure 2E), and the lack of Nrf2 exacerbated this repression at 1, 2, and 5 dpi (Figure 2E). However, there was no change in *Ace2* expression in the BA.4.6 model despite highly increased interferons and inflammatory cytokines (Figure 2F–J). Taken together, the lack of Nrf2 appeared to have a significant impact on lung viral load and/or inflammatory gene expression in mouse models of SARS-CoV-2 infection.

### 3.2. SFN Was Most Effective at Suppressing SARS-CoV-2 among Five Nrf2 Modulators

**Figure 3.**
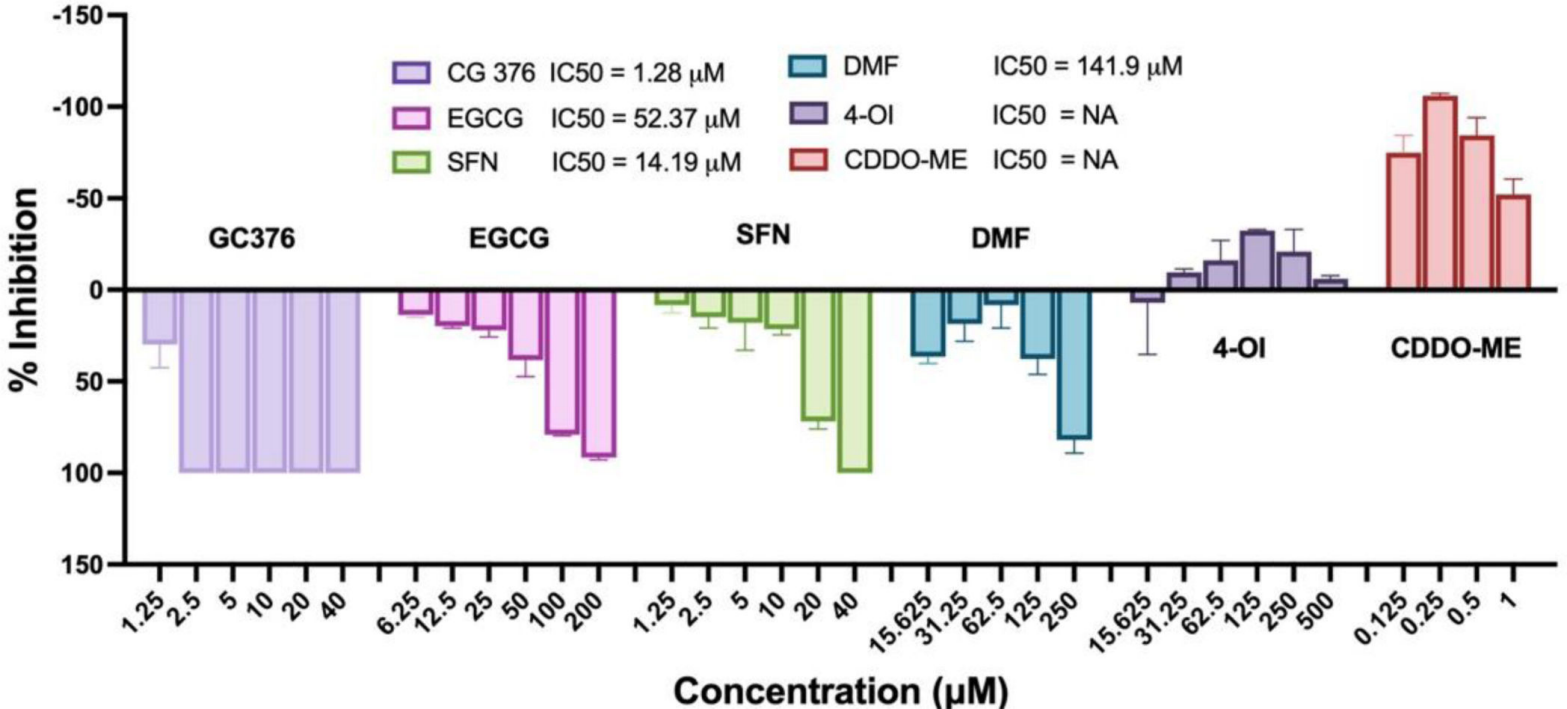
Screening of Nrf2 modulators for their anti-SARS-CoV-2 activities. Vero-E6 cells were pretreated with GC376, EGCG, SFN, DMF, 4-OI, CDDO-ME at different doses as designated in the figure for 1 h, then cells were incubated with 50 PFU SARS-CoV-2 for 1 h followed by continuous treatment of the compounds. Viral production was assayed 72 h later. IC_50_ was calculated as described in Materials and Methods.

To further explore Nrf2’s role in anti-SARS-CoV-2 defense, we evaluated five Nrf2 modulators using the widely used Vero-E6 cell system for anti-SARS-CoV-2 assays. We examined five Nrf2 activators—SFN, EGCG, DMF, CDDO-ME, and 4-OI (Figure 3). As our positive control, we used the common pan-protease inhibitor GC376, which has demonstrated anti-SARS-CoV-2 activity and is currently in clinical trials [30,31]. This choice was also motivated by our initial suspicion, which was later confirmed, that viral proteases might be the potential targets of these compounds. Interestingly, among the Nrf2 activators, SFN, EGCG, and DMF exhibited dose-dependent inhibition of SARS-CoV-2 replication with IC_50_ values of 14.19, 52.37, and 144.20 μM, respectively. However, although other reports indicate antiviral activities of CDDO-ME and 4-OI, we did not observe similar effects in our system. In contrast, both compounds exhibited pro-viral activity, with CDDO-ME being the most potent. Based on these data, we decided to focus on SFN as the most potent antiviral among all the tested Nrf2 activators, showing dose-dependent inhibition. Although GC376 showed higher activity (IC_50_ = 1.28 μM), SFN, with its unique chemical structure and as a natural product frequently used for nutrient supplement, may offer additional benefits for its further development.

### 3.3. SFN’s Antiviral Activity Did Not Act Upon Viral Entry But Targeted the Viral M^Pro^

**Figure 4.**
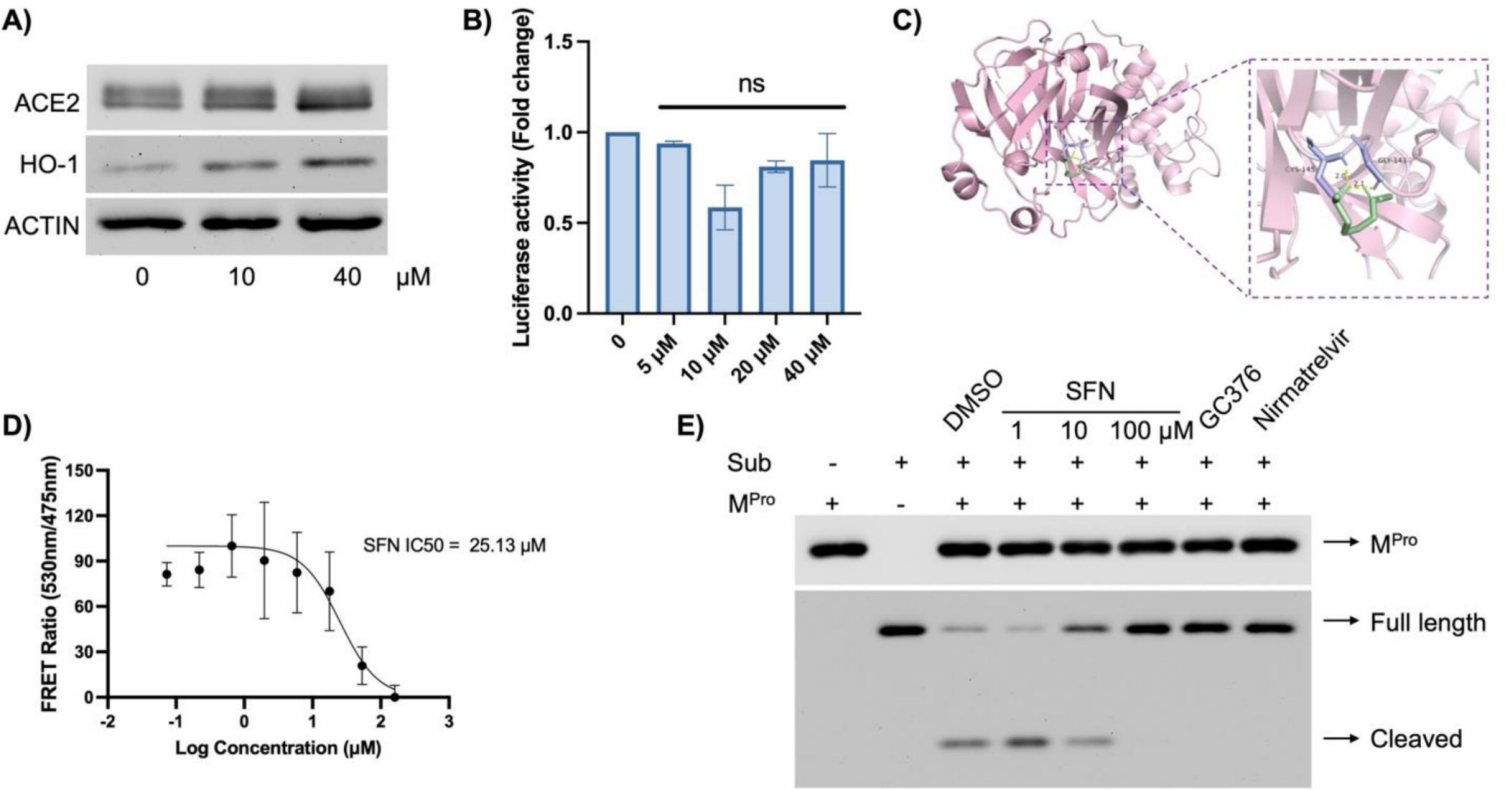
SFN did not affect viral entry but inhibited M^Pro^ activity. (**A**) A549-hACE2 cells were pre-treated with the indicated concentrations of SFN for 1 h, followed by infection with the SARS-CoV-2 at MOI = 1 for 48 h and proteins were collected for the analysis of ACE2, HO-1 and Actin was used as a loading control. HO-1 was tested to show the activation of the NRF2 pathway by SFN. (**B**) A549-hACE2 cells were pre-treated with the indicated concentrations of SFN for 1 h, followed by infection with the SARS-CoV-2 Spike-pseudotyped lentivirus at an MOI of 1. Forty-eight hours later, the cells were lysed and subjected to luciferase activity measurement. (**C**) Autodocking was performed using ADT tools as described in the Materials and Methods. (**D**) FRET analysis using the specific M^Pro^ substrate and purified M^Pro^. (**E**) M^Pro^ protease activity assay. 2 μM M^Pro^ was incubated for 1 h with SFN (1, 10, 100 μM), GC376 (10 μM), Nirmatrelvir (10 μM). DMSO was used as a negative control. Subsequently, the His-tagged substrate was added at a concentration of 5 μM and further incubated for 1 h, followed by Western blot analyses of M^Pro^, intact (full length) and cleaved substrate (Sub).

**Figure 5.**
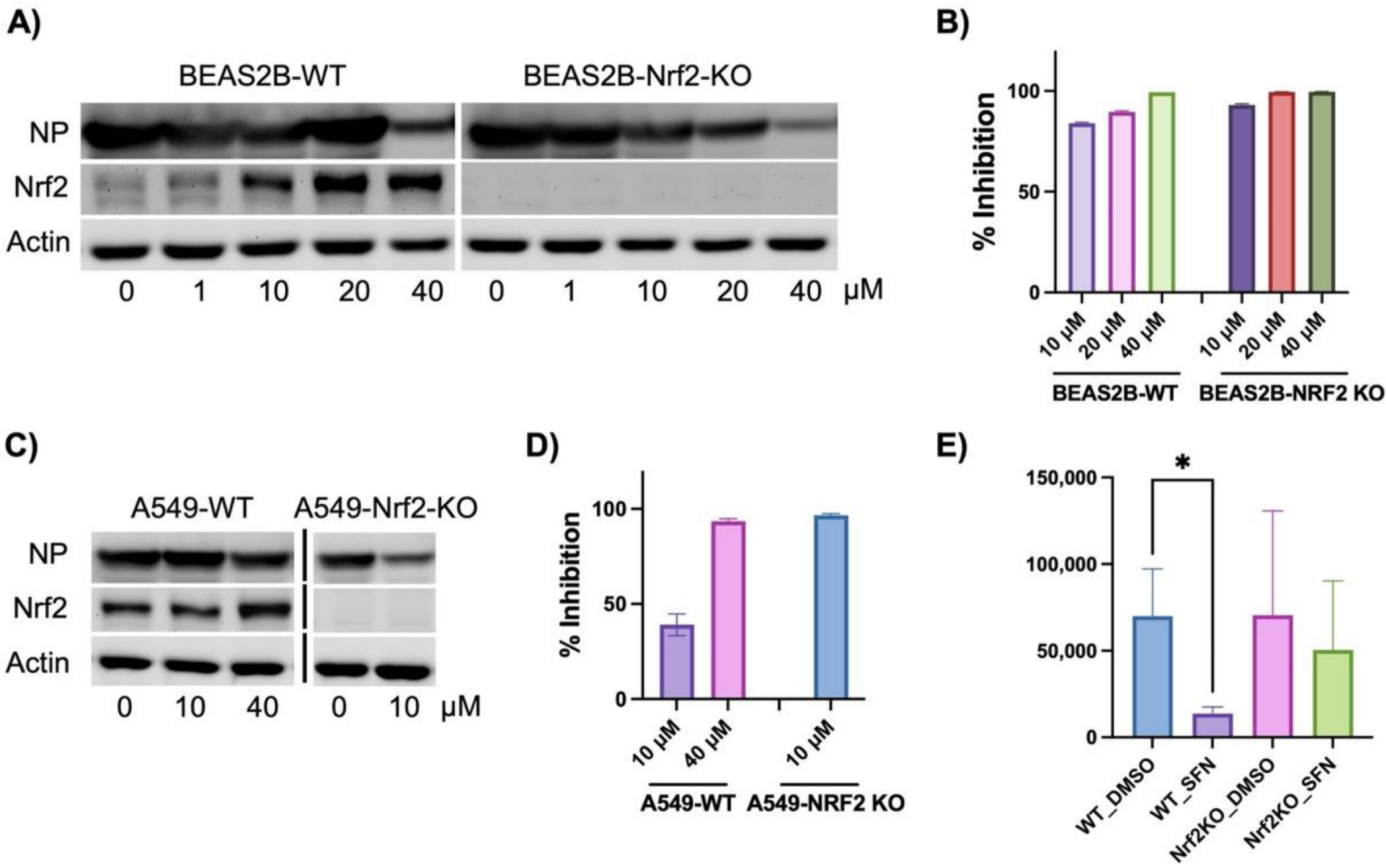
Nrf2-dependent and -independent activity of SFN in inhibiting SARS-CoV-2. Due to the low availability of ACE2 in A549 and BEAS2B cells, all WT and KO cell lines were transduced with adenoviral hACE2 at forty-eight hours before SARS-CoV-2 infections. (**A**,**B**) BEAS2B-WT and BEAS2B-NRF2 KO cells were pretreated with SFN at different doses for 1 h followed by infections with SARS-CoV-2 for 48 h. Total cellular proteins were collected followed by western blot analyses for viral NP and cellular NRF2. ACTIN was used as a loading control. Percentage (%) of inhibition was calculated as following: (1-viral titer (PFU) under SFN treatment/viral tier (PFU) under DMSO treatment) × 100 (%). (**C**,**D**) A549-WT and A549-NRF2 KO cells. The protocols were the same as (**A**,**B**). (**E**) WT and Nrf2 KO mice were pretreated with SFN (10 mg/kg) or the vehicle DMSO one day prior to MA30 infection following by daily treatment of SFN or DMSO. The lung viral titer was collected at 5 dpi. (*n* = 6–8). *: *p* < 0.05.

Next, we investigated the mechanisms through which SFN inhibited viral infection. Due to the low expression of ACE2 in Vero-E6 cells, making it difficult to detect decreased ACE2 expression, we utilized the commonly used cell model A549-hACE2, in which ACE2 was stably expressed in A549 cells. SFN treatment did not appear to affect cellular ACE2 expression (Figure 4A). To interrogate functional relevance, we utilized a pseudovirus model [23], where the SARS-CoV-2 Spike (S) protein was packaged into lentiviral particles to mimic viral infection mediated by the interaction between S and ACE2. The magnitude of successful infection was measured by the activity of luciferase carried by the pseudoviral genome. SFN treatment, across a dose range from 5 to 40 μM, did not affect pseudoviral infection (Figure 4B). Since SFN demonstrated strong anti-SARS-CoV-2 activity in A549 cells (Figure 5C,D below), this data suggests that SFN may not affect viral entry via the S-ACE2 interaction.

To investigate potential mechanisms through which SFN elicited its antiviral function, we performed virtual docking to determine if SFN might interact with viral proteins. Serendipitously, we found that SFN could bind to the M^Pro^ of SARS-CoV-2 (Figure 4C). To verify if SFN could inhibit the protease activity of M^Pro^, we conducted a FRET analysis using a fluorescence-labeled specific peptide substrate of M^Pro^ and purified recombinant M^Pro^. Indeed, we observed dose-dependent inhibition of M^Pro^ activity by SFN, with an IC_50_ of 25.13 μM (Figure 4D). To further validate whether native M^Pro^ exhibited protease activity towards its specific substrate, we incubated purified recombinant M^Pro^ with an M^Pro^ -specific His-tagged substrate and various inhibitors, including SFN, GC376, and Nirmatrelvir (the active component of Paxlovid ^™^). We found that SFN inhibited M^Pro^ dose-dependently by preventing substrate cleavage (Figure 4E). As a positive control, both GC376 [32] and Nirmatrelvir [33] effectively inhibited M^Pro^ activity under the same experimental conditions (Figure 4E). Therefore, M^Pro^ appears to be the target of SFN.

### 3.4. SFN’s Antiviral Activity Was Independent of Nrf2 In Vitro But Required Nrf2 Activity In Vivo

We then tested whether SFN’s antiviral activity depends on Nrf2 activation. To explore this hypothesis, we employed two lung cell lines: A549 and BEAS2B. In addition to WT cells, we also tested cells with endogenous NRF2 deleted using CRISPR technology. Indeed, in both A549-NRF2 KO and BEAS2B-NRF2 KO cells, NRF2 expression was completely eliminated (Figure 5A,C). In WT cells, SFN increased NRF2 protein levels in a dose-dependent manner (Figure 5A,C). The enhancement in BEAS2B cells was more significant than in A549, primarily due to a partial deficiency in KEAP1 in A549 cells, leading to a high baseline NRF2 activation [34]. Nonetheless, both cell models showed complete loss of NRF2 in the KO cells. Despite this deficiency, SFN demonstrated enhanced antiviral activity in both A549-NRF2 KO (Figure 5D) and BEAS2B-NRF2 KO cells (Figure 5B). In A549-NRF2 KO cells, 10 μM SFN was sufficient to completely inhibit SARS-CoV-2 production, while 40 μM was required in A549-WT cells (Figure 5D). A549-NRF2 KO cells could not tolerate the combined treatment of 40 μM SFN and SARS-CoV-2. In BEAS2B-NRF2 KO cells, complete inhibition was achieved at 20 μM SFN, whereas 40 μM SFN was required in WT cells (Figure 5B). The cellular viral protein-NP levels correlated with viral titer under SFN treatment, albeit less sensitive than viral titer in response to SFN treatment (Figure 5A,C). Surprisingly, although SFN exhibited similar antiviral activity in the MA30 model for WT mice as in the cell models, its activity was completely lost in Nrf2 KO mice (Figure 5E). Thus, Nrf2 activity appears to still be required for SFN’s effects under physiological conditions.

## 4. Discussion

Our report is among the first to investigate the causal role of Nrf2 in anti-SARS-CoV-2 infection using *in vitro* cell models. Previous studies on Nrf2 function in SARS-CoV-2 infection have relied either on associative studies using patient biopsies or *in vitro* cell models. The study on patient samples indicating repression of Nrf2 signaling in COVID-19 is highly significant and translational, given its human disease relevance [17]. However, the observation is entirely associative and not causal. The repression of Nrf2 may or may not directly be caused by or have any impact on SARS-CoV-2 infection. Cell studies relying on Nrf2 modulators rarely examined whether their Nrf2 modulating effects indeed mediated the antiviral function. Even if they did, the evidence was often circumstantial. For example, Olagnier et al. demonstrated that Nrf2 activators DMF and 4-OI elicit potent anti-SARS-CoV-2 activity [17]. They attributed this activity to Nrf2 activation by showing that the knockdown of Keap1, an Nrf2 inhibitor, also exhibited antiviral activity. However, this evidence did not support that DMF or 4-OI’s activity was mediated by Nrf2 activation. In fact, we and others [20] have shown that SFN, a potent Nrf2 activator, inhibited SARS-CoV-2 production via an Nrf2-independent mechanism in several cell models using either shRNA knockdown [20] or CRISPR KO approaches (the present study). Similarly, other Nrf2 activators, EGCG [21] and CDDO-ME [22], were also shown to have Nrf2-independent activity. Similar assays can be used to verify if DMF or 4-OI indeed inhibit SARS-CoV-2 production via an Nrf2-dependent mechanism. Along this line, we showed that SFN did not affect viral entry, which is different from the antiviral mechanism of EGCG as it affects viral entry by interacting with ACE2 [21]. Additionally, we showed SFN targets M^Pro^. Interestingly, HO-1, an Nrf2 downstream gene, produces Fe2+ that was speculated to generate Fe2+ that can bind to the divalent metal-binding pocket of the RNA-dependent RNA polymerase (RdRp) of SARS-CoV-2 and inhibit its catalytic activity [35]. However, cell models suffer from high variability. For example, two previously identified anti-SARS-CoV-2 compounds, 4-OI [17] and CDDO-ME [22], exhibited proviral effects in our assay. The discrepancy can be attributed to the use of different cell models. In the previous study [17], 4-OI was tested in highly susceptible VTA cells with over-expressed viral entry factors, ACE2 and TMPRSS2. CDDO-ME was tested in parental Vero and Calu-3 cells [22], both of which expressed low ACE2 compared with the common Vero-E6 cells (an isolate of Vero with high native ACE2 expression) which we used for our screening. Thus, further study is needed to evaluate all those Nrf2 modulators for their anti-SARS-CoV-2 activity across different cell types.

Our report is the first to investigate the causal role of Nrf2 in anti-SARS-CoV-2 infection *in vivo*. Previous studies relied on humanized ACE2 mice [20] or other animal models (e.g. hamsters, ferrets etc. [36]) with scarce gene-targeting capacity, preventing the investigation of the role of Nrf2 in animal models. Our BA.4.6 model has relied on the observation that Omicron variant SARS-CoV-2 can bind mouse ACE2 and directly infect cells [37,38], and we confirmed that mice infected with BA.4.6 showed mild illness without significant weight loss. In contrast, the MA30 model is the most up-to-date mouse model of SARS-CoV-2, mimicking a severe form of the disease [24], and we confirmed that it induced mortality at 5 dpi. In either model, Nrf2 deficiency had a significant, though moderate, impact on lung viral load, viral RNA, and inflammatory gene expressions. Interferon genes were generally correlated with lung viral load in both models. Nrf2 KO mice tended to have higher lung viral load and interferon gene expressions than WT mice. The observation that increased SARS-CoV-2 in Nrf2 KO was not associated with reduced interferons suggests an interferon-independent mechanism may mediate Nrf2’s effects on SARS-CoV-2. Furthermore, inflammatory genes such as *Cxcl10* and *Il-6* were correlated only in the BA.4.6 model. In the MA30 model, however, *Cxcl10* and *Il-6* expression was much lower in Nrf2 KO compared to WT. This observation suggests that Nrf2 deficiency may be somewhat anti-inflammatory in the context of productive SARS-CoV-2 infection, as MA30 was considered to be a replicating virus in mice [24]. This is in direct contrast to the well-established notion of Nrf2’s anti-inflammatory function [5,39], which appears to be manifested in the BA.4.6 model. Whether or not Nrf2 acts differently with respect to its function in SARS-CoV-2 induced airway inflammation will require further study. To showcase the difference between replicating and non-replicating viruses, MA30, but not BA.4.6, was able to repress ACE2 in mice. This is perhaps the first report confirming that downregulating ACE2 at the mRNA level [29,40], the SARS-CoV-2 receptor, also occurred in a mouse model, which provides a valuable *in vivo* model to understand the impact of ACE2 repression on host physiology.

The most striking finding of this study is SFN’s differential dependence on Nrf2 *in vitro* compared to *in vivo*. In the two cell models, SFN’s antiviral activity was unequivocally independent of Nrf2 activation, which aligns with other reports. However, although SFN exhibited potent *in vivo* anti-SARS-CoV-2 activity, its effectiveness required Nrf2. The plausible explanation is that immune cells, which are absent from *in vitro* cell culture since most Nrf2 modulator screenings, including ours, are done in epithelial and stromal cell types, may play a more significant role *in vivo* in the defense against SARS-CoV-2 infection. Indeed, Nrf2 has been established as a key regulator of inflammation and immunity [5,39,41]. Thus, further screening of Nrf2 modulators in antiviral defense may need to include immune cells or more advanced systems such as organoids [42] and organoid co-cultures [43].

## 5. Conclusions

Our study is the first to demonstrate both Nrf2-dependent and independent mechanisms of anti-SARS-CoV-2 activity *in vitro* and *in vivo*.

## Figures and Tables

**Table 1.** List of primers used for qPCR.

Gene	Primer

*β-Actin*	Forward: ACCGTGAAAAGATGACCCAGA Reverse: GGAGTCCATCACAATGCCTGT
*Ifnβ*	Forward: GGCTTCCATCATGAACAACAGGT Reverse: AGGTGAGGTTGATCTTTCCATTCAG
*Ifnλ2/3*	Forward: ACCCTGAAGGTCTGGGAGAAC Reverse: CTGGGAGTGAATGTGGCTCAG
*Cxcl10*	Forward: CTCATCCTGCTGGGTCTGAGT Reverse: CCCTATGGCCCTCATTCTCAC
*Il6*	Forward: AGTTGTGCAATGGCAATTCTG Reverse: CCAGTTTGGTAGCATCCATCA
*Ace2*	Forward: CTGAACACCATGAGCACCATT Reverse: TGTGCTTGTCGCCATTATTTC
*N1*	Forward: GACCCCAAAATCAGCGAAAT Reverse: TCTGGTTACTGCCAGTTGAATCTG
